# Assessing the steroid-sparing effect of biological agents in randomized controlled trials for lupus: a scoping review

**DOI:** 10.1007/s12026-024-09463-y

**Published:** 2024-03-09

**Authors:** Savino Sciascia, Silvia Grazietta Foddai, Marta Arbrile, Massimo Radin, Irene Cecchi, Alice Barinotti, Roberta Fenoglio, Dario Roccatello

**Affiliations:** University Center of Excellence On Nephrologic, Rheumatologic and Rare Diseases (ERK-Net, ERN-Reconnect and RITA-ERN Member) With Nephrology and Dialysis Unit and Center of Immuno-Rheumatology and Rare Diseases (CMID), San Giovanni Bosco Hub Hospital, Department of Clinical and Biological Sciences, University of Turin, 10124 Turin, Italy

**Keywords:** Lupus erythematosus, Biologic therapies, Glucocorticoids, Lupus nephritis

## Abstract

Prompt disease control of flares in patients with systemic lupus erythematosus (SLE) is a priority in treatment strategy planning. However, the long-term dosage-related collateral effects of glucocorticoids (GCs) have pushed researchers towards the identification and utilization of novel biological agents that could both induce and maintain low disease activity and remission, especially in the context of lupus nephritis (LN). This scoping review aims at assessing the current evidence of the potential steroid-sparing effect of biologic therapies by reviewing phase II and phase III randomized, placebo-controlled trials involving SLE/LN patients. A scoping review of the literature was carried out in accordance with PRISMA-ScR recommendations. Risk of bias was assessed through the utilization of the Cochrane Collaboration’s tool for randomized controlled trials (RCTs). Eight RCTs met the inclusion criteria and were included in this analysis (treatment drug, 7 belimumab; 1 anifrolumab). Four studies showed a definite steroid-sparing effect (treatment drug, 3 belimumab; 1 anifrolumab), while in the remaining four RCTs, the steroid-sparing effect was not observed. When focusing on phase III trials, the overall quality of the studies resulted fair or good considering the risk of bias. However, a degree of heterogeneity of steroid regimen protocol (considering initial dosage, tapering and rescue treatment allowance) was observed. While a growing body of evidence is supporting the safety and efficacy of biological treatment in SLE, the evidence on their steroid-sparing effect remains scattered. Future research needs to pursue the identification of precise SLE clusters of patients who would benefit most from a specific treatment protocol with a definite steroid-sparing effect.

**Supplementary Information:**

The online version contains supplementary material available at 10.1007/s12026-024-09463-y.

## Introduction

Prompt disease control of flares in patients with systemic lupus erythematosus (SLE) is a priority in treatment strategy planning. Glucocorticoids (GCs) have always been used by physicians to treat lupus patients to obtain swift disease control; however, due to the high burden of short- and long-term dosage-related collateral effects, the feasibility of alternative, steroid-sparing treatment approaches has been intensely investigated.

In the last decade, we have witnessed a growing number of studies exploring the safety and efficacy of novel biological agents for both inducing and maintaining low disease activity and remission, especially in the context of lupus nephritis (LN), which is one of the most common organ-related complications of SLE. While biologics are opening a new era for SLE/LN management, clear evidence supporting their net benefit as GCs-sparing agents is still lacking.

Due to these facts, it would be useful to explore the recent evidence on the potential steroid-sparing effect of biologic therapies currently used to treat SLE patients, especially those affected by LN, in phase II and phase III randomized, placebo-controlled trials (RCTs). Similarly, it might be worth exploring unmet needs in available studies, especially from a methodological perspective. To answer these questions, we designed a scoping review to synthesize the available evidence, while highlighting points of improvement that could be addressed by future researches [1].

## Methods

### Protocol and registration

To conduct this scoping review, we adhered to the guidelines outlined in the Preferred Reporting Items for Systematic Reviews and Meta-Analysis extension for scoping reviews (PRISMA-ScR) [2]. Although a protocol was devised, it was not formally registered.

We formulated two key research questions:

1. What insights does the literature provide regarding the steroid-sparing effect of biological agents in individuals with SLE?
2. Can we substantiate a clear steroid-sparing effect associated with any specific biologic treatment utilized in the available SLE trials?

### Eligibility criteria and search strategy

Trials involving patients with SLE, regardless of age, were identified from the MEDLINE (via PubMed) and EMBASE databases on April 18, 2022, using the following key search strategy (as detailed in the supplementary material S1). Search results were confined by using PubMed filters to human clinical trials in English, with full-text articles available for review (excluding conference abstracts alone). Inclusion criteria, following JMB recommendations for scoping reviews, encompassed the population (phase II or III trials involving SLE patients, with or without LN), concept (studies providing detectable steroid data indicating the potential for biological treatments to exhibit a steroid-sparing effect) and context (studies applying biological treatments). The steroid-sparing effect was defined as the ability of the investigated immunosuppressant regimen to reduce both the daily dose and the cumulative prednisone dosage compared to standard treatment at the study’s conclusion. Studies that did not meet these criteria were explicitly excluded from the research. The results of the search and study inclusion process are depicted in a PRISMA-ScR flowchart (Supplementary 2). The risk of bias was assessed using the Cochrane Collaboration’s tool for RCTs [3].

### Data extraction

Studies that met the inclusion criteria were independently reviewed for eligibility confirmation based on title and abstract by two researchers (SS and SGF). In case of disagreement, a third researcher (MR) was consulted to reach a consensus in the evaluation stage. Data items were defined and extracted independently by the two researchers (SGF and SS), while data registration in an Excel Table (Microsoft, Redmond, 110 WA, USA) resulted after disagreement discussion and identification of consensus. As the data on eligibility were dichotomous (eligible: yes/no), agreement at both the title and abstract review and the full article review stages was determined by calculation of Cohen’s kappa coefficient (*k* > 0.8). If consensus could not be achieved, a third part (MR) cleared up the disagreement.

Extracted data included pertinent details about the participant, the concept, the context and the key findings relevant to the review questions.

After reaching a consensus on papers selection and data extraction, three analyses were conducted:

1. Line I data analysis: included both phase II and phase III studies to evaluate the whole impact of steroid-sparing therapies on GC utilization (considered both as cumulative dosage and as for dosage at the end of the study follow-up);
2. Line II data analysis: compared GC tapering potential of the counterposed immunosuppressant therapies in phase II and phase III trials in LN cohorts;
3. Line III: evaluated the quality of evidence of phase III RCTs (clean data analysis) considering both patients with and without LN.

Cochrane Risk of Bias Tool for RCTs was applied to papers selected for the clean data analysis and for the sub-analysis. Bias assessment was independently evaluated and then critically discussed by the two researchers (SGF and SS). When consensus on one of the defined items was not reached, a third part (MR) resolved the disagreement.

## Results

As a result, a total of 30 articles were identified through the literature search. During the data analysis phase, eight RCTs met the inclusion criteria [4–11]. In summary, seven studies evaluated B-cell-targeted therapies [4, 5, 7–11], while one study investigated the use of anifrolumab [6]. Table 1 provides an overview of study populations, biological treatments and the presence or absence of a net steroid-sparing effect. Detailed treatment regimens and trial results are outlined in Table 2. Supplementary Table 2. Supplementary 2 presents the screening process in a PRISMA flowchart.

**Table 1:**
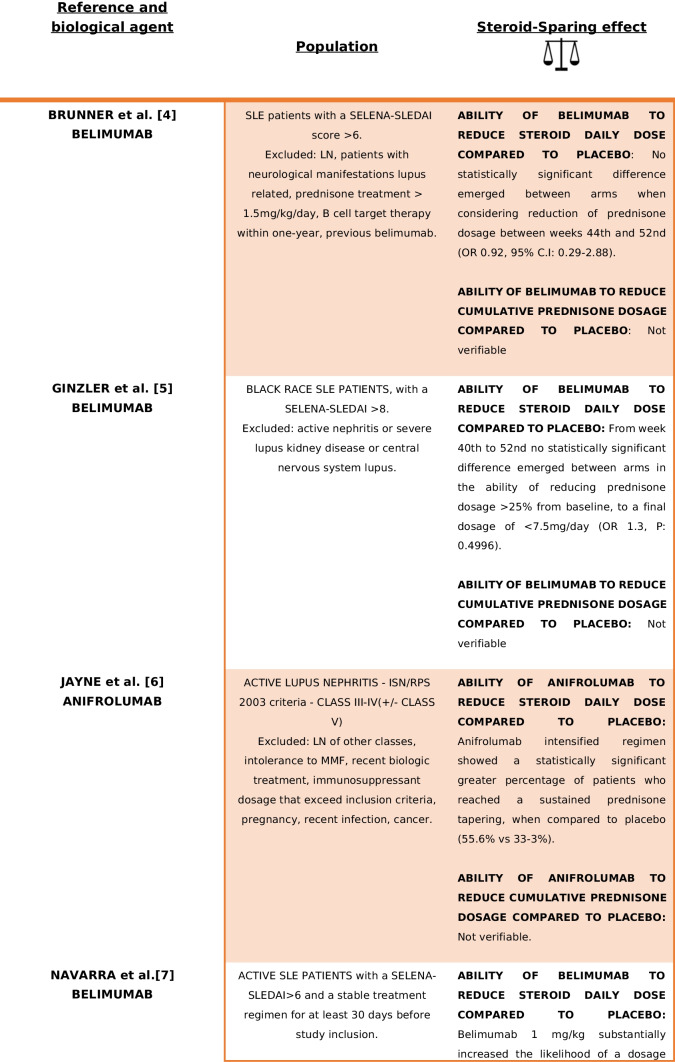

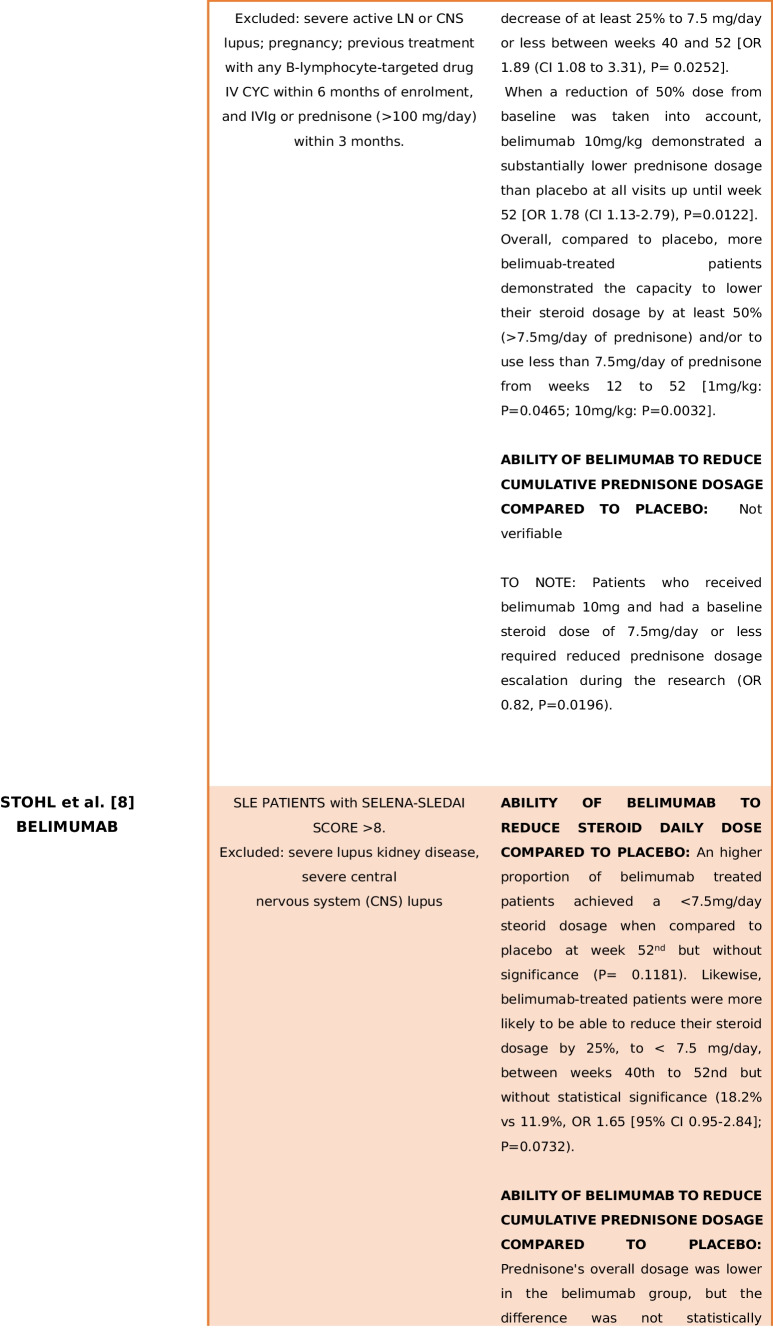

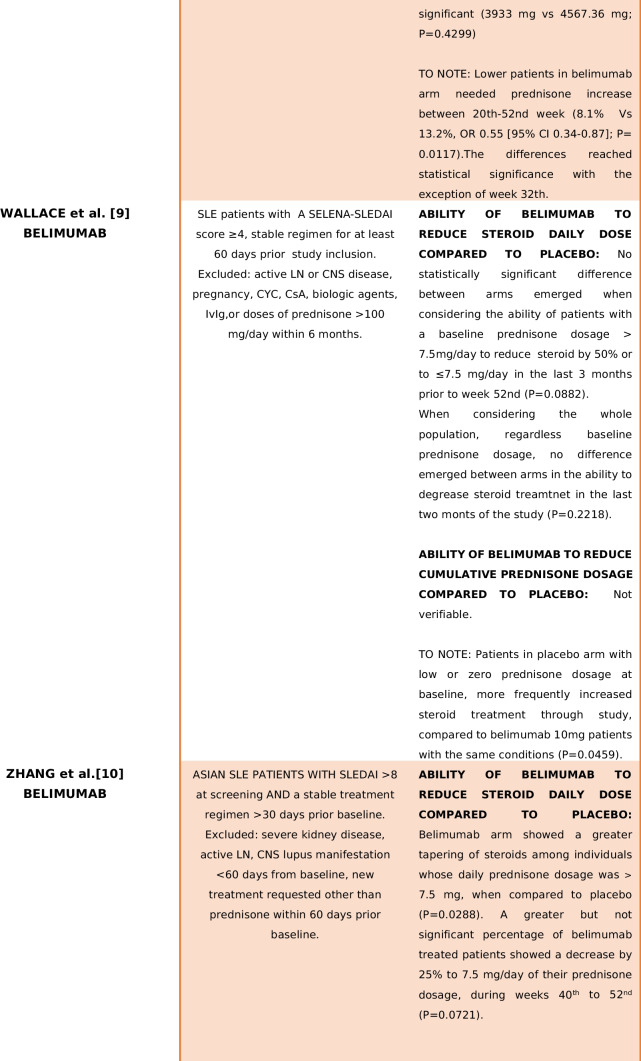

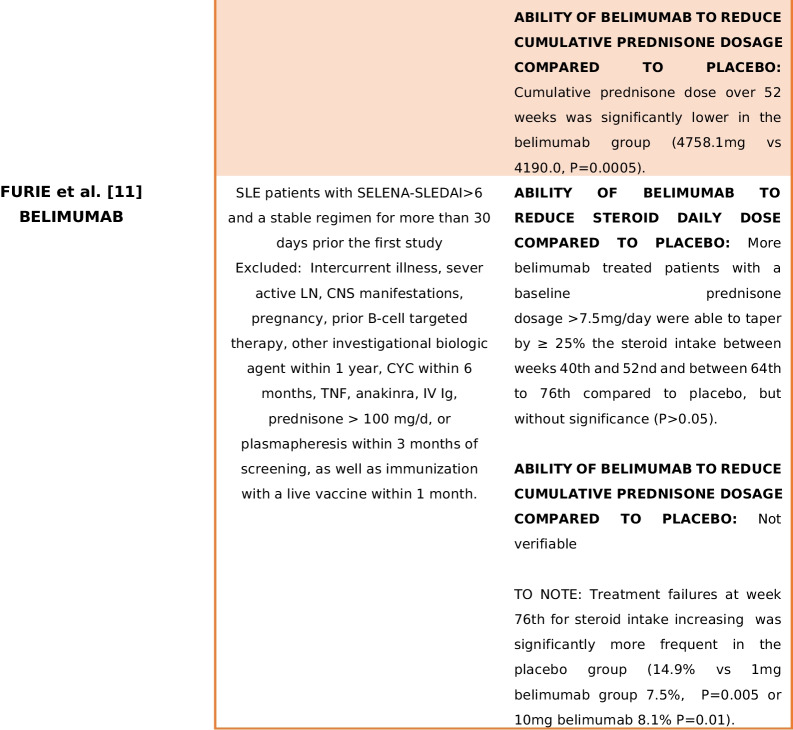
Main findings in terms of GC-sparing effect of the trials included in the analysis

Summarize the type of population, biological treatment and the presence or absence of a net steroid-sparing effect.

*CYC* cyclophosphamide, *CsA* cyclosporin A, *TNF* tumour necrosis factor, *NSAID* non-steroid-sparing agent, *CNS* central nervous system, *LN* lupus nephritis

**Table 2:** Main characteristics of the trials included in the analysis

*First author and study identification name/code*	*Population*	*Treatment*	*Glucocorticoid dosage*	*Results*
Brunner [4] PLUTO	5–17 years old SLE patients (per ACR criteria) with SELENA-SLEDAI score > 6	Belimumab 10 mg + SoC vs placebo + SoC (including immunosuppressant and corticosteroids)	No forced steroid tapering, but if a clinically significant improvement was assessed by clinicians for more than 4 or 12 weeks (in case of unstable clinical situation) consecutively, prednisone dosage could be progressively reduced to < 7.5 mg/day	At baseline median (IQR) corticosteroid dose was lower in the belimumab group (7.5 mg vs 10 mg/day)
	Stable treatment regimen with glucocorticoids, NSAIDs, antimalarials or immunosuppressive treatments for ≥ 30 days before study initiation	*Allowed treatment modifications*: Immunosuppressant might increase until the 16th (with specified maximum dosage/treatment); after that time, it should be maintained at the baseline-16th weeks dosage or reduced; antimalarials could increase or started until 16th weeks, after the same rules present for immunosuppressants applied	Prednisone tapering was considered a secondary efficacy endpoint	Between weeks 44th and 52nd, 20.0% of belimumab and 21.1% of placebo patients had ≥ 25% reduction in baseline prednisone dosage from baseline (OR 0.92, 95% CI 0.29–2.88)
	*Exclusion criteria*: Active LN and/or neurological manifestations lupus related, prednisone treatment > 1.5 mg/kg/day, B cell target therapy within 1-year, previous belimumab administration	Steroids could be increased but needed to return to baseline dosage plus 25% or should be as the baseline dosage plus 5 mg of steroid in the final dosage until week 44th. Until week 44th, intra-articular injections were allowed. Patients could receive topical steroid for a defined time for non-SLE-related reasons		Median corticosteroid doses did not decrease by week 52nd in either of the treatment groups
		*Not allowed*: Augmentation of baseline prednisone dosage or 44th week prednisone dosage from week 44th to 52nd		
Ginzler [5] EMBRACE	Black race SLE Patients, age > 18 years old, with a SELENA-SLEDAI score ≥ 8	Two study arms: belimumab 10 mg + SoC vs placebo + SoC	No forced steroid tapering was applied, but prednisone dosage decreasing was considered a secondary endpoint	From weeks 40th to 52nd, 14.7% patients from belimumab group and 12.6% from placebo group achieved a prednisone reduction > 25% from baseline to a final dosage of < 7.5 mg/day (OR 1.3, *P* 0.4996)
	*Exclusion criteria*: previous treatment with belimumab, active nephritis or severe lupus kidney disease or central nervous system lupus	SOC included: steroids, antimalarials, NSAIDs and immunosuppressive therapies with the exception for biologics and IV CYC treatments	At baseline, 17.7% of belimumab-treated patients vs 14.8% of placebo arm were not under steroid treatment; 20.7% vs 21.5% were taking ≤ 7.5 mg/day, while 61.5% vs 63.8% were under > 7.5 mg/day of prednisone respectively	In the open-label extension phase, almost 32% of the continuous belimumab treatment arm achieved a prednisone reduction < 7.5 mg/day compared to 14.8% of placebo arm patients that started belimumab in the second phase of the study
Jayne [6] TULIP-LN	18–70 years old patients with a recent (3 months maximum) biopsy proven diagnosis of classes III–IV (± class V) active lupus nephritis (ISN/RPS 2003 criteria), high dsDNA and/or anti-SM antibodies, UCPR > 1 mg/mg and eGFR > 35 ml/min	Three study arms: two with different anifrolumab dosage (300 mg/900 mg), one placebo. All arms received standard treatment with MMF (2 g/day from weeks 8th to 52nd) and PDN (before randomization maximum dosage allowed: 40 mg/day	All subjects received an IV methylprednisolone pulse (500 mg) within 10 days of randomization or at randomization	Sustained prednisone tapering (prednisone < 7.5 mg/day) was similar in anifrolumab basic regimen and placebo (35.5% vs 33.3%), but greater in intensified anifrolumab group (55.6% vs 33.3%)
	Screened population was formed by 26.9% class III LN(41% with concomitant class V), 73.1% class IV LN (with 21.7% with concomitant class V LN)	*Allowed treatment modifications*: (1) Decrease of MMF < 1 g/day for less than 14 days or in case of low body weight; increase of MMF to 3 g/day until week 24th with decrease to 2 g/day by week 32th	Oral glucocorticoid dosage tapering was mandatory to be < 10 mg/day by week 12th and < 7.5 mg/day by week 24th	
	*Exclusion criteria*: other class of LN not included in the inclusion criteria or concurrent renal disorder that might interfere with the provided treatment; intolerance to MMF; pregnancy or recent pregnancy complication; one of the following treatment at higher dosage than the one described (steroids OS > 0.5 mg/kg 7-day IV steroids > 3 g; IV CYC 2 pulses at high dose or 4 pulses at low dose; MMF > 2.5 g/day > 8 weeks); tacrolimus > 4 mg/day > 8 weeks; specified B cell targeted therapy restrictions that vary in relation to administered molecule; receipt of experimental/biologic treatment within 4 weeks/5 half-lives; lack of discontinuation of the following treatment before randomization: MTX, AZA, TAC, CsA, CYC, INF therapy leflunomide; recent administration of vaccine (live/attenuated), comorbidities: NP-SLE, suicidal ideation, SSc, CAPS, APS, IDs, HIV, HBV, HCV, HZV, HSV, CMV, recent infection, cancer	(2) One additional methylprednisolone pulse (≤ 500 mg or split over 2 consecutive days) allowed between weeks 0 and 8th		
		(3)Temporary increasing of prednisone dosage to a maximum of 40 mg/day for ≤ 14 days and by day 15th tapering to equal or less dosage than before prednisone augmentation OR one intra-articular injection of PDN ≤ 80 mg NOT beyond 40th week		
		(4) HCQ, statins and antihypertensive treatment could change during weeks		
		*Not allowed*: Treatments and regimens not included among allowed medications		
Navarra [7] BLISS-52	Patients > 18 years old affected by SLE (per ACR criteria), with SELENA-SLEDAI score > 6 and a stable treatment regimen for at least 30 days before the study enrolment	Three treated arms: belimumab 1 mg/10 mg + SoC vs placebo + SoC through week 52nd	No prednisone restriction dosage until 24 weeks, but the proportion of patients with an average reduction of steroid dosage of 25% from baseline to ≤ 7·5 mg/day during weeks 40th to 52nd was considered a secondary endpoint	69% of patients were taking prednisone at doses greater than 7.5 mg/day at baseline
	*Exclusion criteria*: severe active LN or CNS lupus; pregnancy; previous treatment with any B-lymphocyte-targeted drug or IV CYC within 6 months of enrolment and IVIg or prednisone (> 100 mg/day) within 3 months previous study initiation	*Allowed treatment modification*: SoC modification was allowed until week 16th for immunosuppressive drugs and until 24th week for antimalarial drugs	Tapering protocol was decided by clinicians	A reduction in dose of at least 25% to 7.5 mg/day or less between weeks 40th and 52nd was significantly greater with belimumab 1 mg/kg (OR 1.89 (CI 1.08 to 3.31), *P* = 0.0252)
		Prednisone could be increased in dosage but needed to return to within 25% or 5 mg greater than the baseline dose by 24th week		Belimumab 10 mg/kg showed a significantly reduction in prednisone dosage at all visits until week 52nd compared to placebo, when a reduction of ≥ 50% dosage form baseline was considered (OR 1.78 (CI 1.13–2.79), *P* = 0.0122)
				Patients treated with belimumab 10 mg, who had a baseline steroid dosage of ≤ 7.5 mg/day, needed less prednisone incrementation during study (OR 0.82, *P* = 0.0196)
		*Not allowed*: Addition of a new immunosuppressant or biologic treatment at any time. Initiation of a new antimalarial drug or angiotensin-converting enzyme inhibitors or statins could be initiated after 4 and 6 months respectively		Belimumab patients showed a significant sustained reduction of prednisone dosage (defined as the decrease of at least 50% of the baseline dosage > 7.5 mg/day and/or a dosage < 7.5 mg/day of prednisone from week 12th to week 52nd) in comparison to placebo [1 mg/kg: *P* = 0.0465; 10 mg/kg: *P* = 0.0032]
Stohl [8] BEL112341	Patients > 18 years old affected by SLE (per ACR criteria) with a SELENA-SLEDAI score > 8	Two study arms: Belimumab 200 mg sc + SoC vs placebo + SoC	Glucocorticoid tapering was not forced, however a steroid 25% reduction from baseline to < 7.5 mg/day between 40–52nd week (in those patient with a baseline prednisone dosage of more than 7.5 mg/day) was considered a secondary endpoint	A higher proportion of patients in the belimumab group had their corticosteroid dosage decreased from 7.5 mg/day at baseline to < 7.5 mg/day at week 52nd, although this did not achieve statistical significance (*P* = 0.1181)
	Patients must have received a stable treatment for at least one moth prior to enrolment	SOC included: steroids, antimalarials and immunosuppressive therapies	At baseline 60.2% of patients were receiving > 7.5 mg/day prednisone (335 in belimumab group, 168 in the placebo)	Lower patients in belimumab arm increased prednisone between 20th and 52nd weeks (8.1% vs 13.2%, OR 0.55 [95% CI 0.34–0.87]; *P* = 0.0117).The differences were significant from week 20 to week 52nd, except for week 32nd
	*Exclusion criteria*: Severe lupus kidney disease, severe central nervous system (CNS) lupus			When compared to placebo-treated patients, belimumab-treated patients were more likely to be able to reduce their corticosteroid dosage by 25%, to < 7.5 mg/day, between weeks 40th to 52nd (18.2% versus 11.9%), although this difference did not achieve statistical significance (OR 1.65 [95% CI 0.95–2.84]; *P* = 0.0732)
				Prednisone's overall dosage was lower in the belimumab group, but the difference was not statistically significant (3933 mg vs 4567.36 mg; *P* = 0.4299)
Wallace [9] NTC00071487	Patients > 18 years affected by SLE (per ACR criteria) with a SELENA-SLEDAI score ≥ 4 and a stable treatment regimen for at least 60 days prior study inclusion	Two study arms: belimumab 1 mg/4 mg/10 mg + SoC vs placebo + SoC	Glucocorticoid tapering was not forced, but the percentage of patients with steroid dosage < 7.5 mg/day or reduced by 50% from baseline during weeks 40th–52nd was considered a secondary endpoint	There was not a statistically significant difference among groups in percentage of patients, whose baseline prednisone dose was > 7.5 mg/day and were able to reduce their steroid dose by 50% or to ≤ 7.5 mg/day in the last 3 months prior to the week 52 visit (*P* = 0.0882)
	*Exclusion criteria*: active LN or CNS disease, pregnancy, treatment with cyclosporine, IvIg, biologics, CYC or doses of prednisone > 100 mg/day within 6 months prior study inclusion	Unlimited changes in prednisone and immunosuppressive medications were allowed during the trial		In the last 2 months of study, prednisone dosage reduction among groups was not different (*P* = 0.2218)
				However, in patients on either no steroids or low-dose steroids (≤ 7.5 mg/day) at baseline, prednisone was statistically more frequently increased to > 7.5 mg/day among placebo patients, compared to belimumab 10 mg (*P* = 0.0459)
Zhang [10] BEL113750	Patients > 18 years old affected by SLE (per ACR criteria) with a SLEDAI score > 8	Two study arms: belimumab 10 mg + SoC vs placebo + SoC	No forced tapering was requested, but (1) Number of days of daily prednisone dose ≤ 7.5 mg and/or reduced by 50% from baseline over 52nd weeks in patients with baseline dose > 7.5 mg/day	Among patients with a baseline prednisone dose of > 7.5 mg/day, there was a significant reduction in steroid use favouring belimumab (*P* = 0.0288)
	Patients required a stable treatment regimen for at least 1 month prior enrolment	Unlimited changes in prednisone and allowed immunosuppressive medications (as per inclusion	(2) Cumulative prednisone dose over 52nd weeks	Belimumab-treated patients had a longer duration of reduced steroid use, compared with placebo
	*Exclusion criteria*: Severe kidney disease, active LN and/or CNS lupus manifestation sixty days from baseline, new treatment initiation (B-cell therapy at any time; 1-year prior biologic therapy; 3 cycles of oral or intravenous steroid usage for non-SLE related causes in the last year; CYC treatment 180 days before enrolment) — aside from prednisone — within 60 days prior baseline, cancer history, immunodeficiency, severe infections, uncontrolled chronic disorders non-SLE related	criteria) were allowed during the trial	(3) The percentage of patients whose average prednisone dose was reduced by ≥ 25% from baseline to ≤ 7.5 mg/day between weeks 40th and 52nd and were considered as secondary end point	Cumulative prednisone dose over 52 weeks was significantly lower in the belimumab group (4758.1 mg vs 4190.0, *P* = 0.0005)
				More patients in the belimumab group had a dose reduction of ≥ 25% to ≤ 7.5 mg/day during weeks 40th–52nd, but the difference was not statistically significant (*P* = 0.0721)
Furie [11] BLISS-76	Patients > 18 years old affected by SLE (per ACR criteria), with a SELENA-SLEDAI score > 6 and a stable treatment regimen (antimalarials, steroids, immunosuppressant therapies) for more than 30 days prior the study inclusion	Three treated arms: belimumab 1 mg, belimumab 10 mg vs placebo through week 72th	No forced tapering was requested, but the percentage of patients with a mean prednisone dose decrease ≥ 25% from baseline and a final dose of ≤ 7.5 mg/day during weeks 40th to 52nd was considered a secondary endpoint of the study	A subgroup of 376 patients (46%) was receiving prednisone > 7.5 mg/day at baseline
	*Exclusion criteria*: Intercurrent illness, sever active LN, CNS manifestations, pregnancy, prior B-cell targeted therapy, other investigational biologic agent within 1 year, CYC within 6 months, TNF, anakinra, IVIg, prednisone > 100 mg/day or plasmapheresis within 3 months of screening, as well as immunization with a live vaccine within 1 month	*Allowed treatment modifications*: Until week 16th, antimalarials and immunosuppressive drugs dosage could change	Steroid tapering proceeded at clinician discretion	A greater proportion of belimumab-treated patients reduced prednisone by ≥ 25% and to ≤ 7.5 mg/day between weeks 40th and 52nd and between 64 and 76th weeks compared to placebo, but without significance (*P* > 0.05)
		Any steroid dosage modification was allowed until week 24th		Treatment failures at week 76th for a not allowed prednisone dosage was significantly more frequent in the placebo group (14.9% vs 1 mg belimumab group 7.5%, *P* = 0.005 or 10 mg belimumab 8.1% *P* = 0.01)
		*Not allowed*: The initiation of a new immunosuppressive, inhibitor of the renin-angiotensin system or biologic therapy; treatment dosage augmentation (immunosuppressive o antimalarials) greater than the one present at baseline or at week 16th		There was also a trend toward a greater proportion of patients treated with placebo increasing their prednisone dosage between 16 and 76th weeks when compared to belimumab arms, but without reaching significance
		From weeks 24th to 44th, prednisone dosage should not be 5 mg or 25% higher than baseline		
		After week 44th, steroid dosage should not exceed baseline or 44th week dosage		

*CYC* cyclophosphamide, *CsA* cyclosporin A, *TNF* tumour necrosis factor, *NSAID* non-steroid-sparing agent, *RTX* rituximab, *MMF* mycophenolate mofetil, *PDN* prednisone, *IvIg* intravenous immunoglobulin, *AM* antimalarial, *IS* immunosuppressive, *CNS* central nervous system, *LN* lupus nephritis

In the TULIP-LN trial conducted by Jayne et al., the biologic utilized was anifrolumab [6]. The trial explicitly outlined a precise GCs regimen from the study’s outset, applying to both the placebo and treatment arms. This regimen involved methylprednisolone infusion (500 mg IV, within 10 days of randomization or at randomization) and/or oral administration, with a corresponding meticulous tapering schedule (mandatory dosage: 10 mg/day by week 12th; < 7.5 mg/day by week 24th). While a sustained prednisone tapering was similar in the anifrolumab basic regimen and placebo, a higher proportion of intensified anifrolumab patients demonstrated a distinct steroid-sparing effect. This effect, defined as the treatment’s ability to reduce daily steroid intake (55.6% vs. 33.3%), was more pronounced compared to the placebo [6].

In the EMBRACE (Ginzler et al.) and PLUTO (Brunner et al.) trials [4, 5], both investigations centred on the utilization of belimumab. Steroid treatment was permitted across all arms throughout the analysis weeks, and no mandatory steroid tapering was prescribed. However, the assessment of prednisone dosage reduction was considered in the final analysis, revealing no discernible differences in steroid consumption between patients treated with biologics and those receiving standard care. Specifically, in the Ginzler trial [5], during weeks 40 to 52, 14.7% of patients in the treated arm compared to 12.6% in the placebo arm achieved a prednisone reduction exceeding 25% of the initial dosage and/or a daily steroid intake < 7 mg/day (OR 1.3, *P* = 0.4996). In the Brunner study [4], between the 44th and 52nd weeks, 20% of belimumab-treated and 21.1% of placebo patients experienced a ≥ 25% reduction in prednisone baseline dosage (which was lower at baseline in the belimumab-treated arm, OR 0.92, 95% CI 0.29–2.88), while the median GCs dosage did not decrease by week 52 in either arm. In BEL113750 (Zhang et al.) trial [10], though no forced prednisone tapering was requested, cumulative steroid dosage and tapering maintenance were considered as secondary endpoints. Belimumab population showed a significant reduction in the cumulative steroid dosage undertaken by patients in the 52 weeks of observation (4758.1 mg vs 4190.0 mg, *P* = 0.0005), a more stable and tight prednisone decrease when compared to placebo (40th–52nd weeks prednisone tapering ≥ 25% in relation to baseline and/or ≤ 7.5 mg/day (15.6% vs 10.9%; OR 1.68, *P* = 0.0721); daily dose reduction for patients with baseline steroid intake > 7.5 mg/day favours belimumab (*P* = 0.0288)).

In BEL112341 (Stohl et al.) trial [8], the studied biologic was belimumab. In detail, a steroid tapering regimen was not forced, but steroid dosage was considered as a secondary endpoint. The belimumab arm showed a greater reduction in prednisone dosage among weeks and/or less dosage adjustments when compared to placebo (with significance between weeks 20th and 52nd except for week 32nd (8.1% vs 13.2%, OR 0.55 [95% CI 0.34–0.87]; *P* = 0.0117). Indeed, the whole cumulative dose of prednisone in the belimumab arm resulted to be lower, but without reaching significance when compared to placebo (3933 mg vs 4567 mg; *P* = 0.4299).

In the BLISS-76 trial conducted by Furie et al. [11], investigating the use of belimumab, any prednisone dosage was permissible through week 24. Subsequently, only temporary and well-defined adjustments to steroid intake were allowed; however, prednisone reduction was at the investigator’s discretion. The placebo group experienced more treatment failures (14.9% vs. 7.5%, *P* = 0.005 or 8.1%, *P* = 0.01) and prednisone dosage adjustments between weeks 16 and 76, although statistical significance was not reached. Additionally, a greater proportion of belimumab-treated patients reduced prednisone by ≥ 25% and to ≤ 7.5 mg/day between weeks 40 and 52 and between weeks 64 and 76 compared to the placebo, but these differences were not statistically significant (1 mg belimumab: 19%, 10 mg belimumab 18%; placebo 13%,* P* > 0.05).

Data from the BLISS-52 trial (Navarra et al.) [7], which investigated belimumab and specified the same restriction in steroid usage from week 24, indicated a significant superiority in both overall prednisone dosage reduction (10 mg/kg belimumab at 52 weeks, OR 1.78 (CI 1.13–2.79), *P* = 0.0122) and sustained steroid tapering (tapering for more than 12 weeks, 1 mg/kg belimumab, *P* = 0.0465; 10 mg/kg belimumab, *P* = 0.0032) in the belimumab-treated arms compared to the placebo. In the trial NTC00071487 by Wallace et al. [9], exploring the utilization of belimumab, unlimited changes in prednisone and immunosuppressive medications were allowed, but prednisone dosage reduction was considered a secondary endpoint. No difference emerged between groups in prednisone reduction; however, placebo patients with no steroid or low-dosage steroid at the beginning of the study manifested a higher increase in prednisone dosage compared to the belimumab-treated arm (*P* = 0.0459).

Lastly, as previously mentioned, a sub-analysis was carried out considering only those papers assessing the GCs sparing effect in patients with LN (both considering phase II and phase III trials). Only one paper [6] was eligible for the analysis. In TULIP-LN trial [6], even though all included patients received one intravenous methylprednisolone pulse before randomization and a mandatory regimen at weeks 12th and 24th (< 10 mg/day and < 7.5 mg/day respectively), intensified anifrolumab arm showed greater sustained steroid reduction in comparison to other arms. It is worth noting, however, that the placebo group had better renal response and GCs tapering maintenance in comparison to basic anifrolumab regimen.

### Quality of the evidence

When specifically assessing phase III trials through a meticulous data analysis [5, 7–11], encompassing both LN and SLE patients without LN, the overall quality of the studies was deemed fair or good, as outlined in Table 3, considering the risk of bias. In particular, four studies [5, 7, 10, 11] exhibited a low risk of bias in various aspects, including random sequence generation, allocation concealment, blinding procedures, data outcomes, selective reporting and other potential biases. However, in the trials led by Stohl and Wallace [8, 9], there is no explicit statement regarding the methodology applied in random sequence generation, allocation concealment and blinding of personnel and participants. Nevertheless, a thorough discussion of all remaining biases is presented, and in conclusion, there is no solid data supporting the notion that the validity of results is less than fair.

**Table 3:** Cochrane Risk Bias Tool for randomized controlled trials

								Overall standard
Ginzler [5] EMBRACE	L	L	L	L	L	L	L	Good
Navarra [7] BLISS-52	L	L	L	L	L	L	L	Good
Stohl [8] BEL112341	U	U	U	L	L	L	L	Fair
Wallace [9] NTC00071487	U	U	U	L	L	L	L	Fair
Zhang [10] BEL113750	L	L	L	L	L	L	L	Good
Furie [11] BLISS-76	L	L	L	L	L	L	L	Good
	Random sequence generation	Allocation concealment	Blinding of participants and personnel	Blinding of outcome assessment	Incomplete outcome data	Selective reporting	Other bias	

*L* low risk for bias, *H* high risk for bias, *U* uncertain risk for bias

## Discussion

The utilization of biological treatments demonstrated a clear steroid-sparing effect in five investigations, comprising four RCTs focusing on belimumab [8, 10, 11] and one on anifrolumab [6]. What insights can be gleaned from this analysis? Firstly, a steroid-sparing effect is achievable with various treatments. Secondly, to accurately identify and assess a drug’s potential to reduce the cumulative dose of prednisone per patient, certain steps are crucial:

- establish a consensus on the definition of a steroid-sparing effect;
- advocate for the inclusion of the steroid-sparing effect as an outcome in all future drug efficacy studies involving SLE patients.

### Strengths and limitations

While the novelty of our scoping review lies in attempting to address an unmet need for patients with SLE and LN, we acknowledge several limitations that affect the robustness of our conclusions. Firstly, the identified trial protocols exhibit heterogeneity, and the inclusion of a strong standard of care regimen with a robust steroid supplement in the early phase of the trial may have obscured the effective and beneficial role of the new drug in reducing overall GCs consumption, leading to a significant knowledge gap.

Furthermore, steroid dosage adjustments were permitted in all studies at various time points and dosages, contributing to the potential impact of prednisone on the efficacy of immunosuppressive treatment. The choice of a permissive complementary treatment was influenced by the characteristics of the population under examination and the ethical imperative of preserving the patient’s health. Active SLE patients, especially those with LN or refractory LN, require prompt disease control, explaining the fluctuation in steroid regimens, which are still considered rescue treatments in SLE management.

Considering all these points, it is challenging to provide clear indications on when and if it is possible to consider these described molecules as “steroid-sparing” agents. It may be more accurate to currently view these drugs as valuable add-on therapies to pre-existing standard of care treatments. In phase III trials on LN, the complete steroid-sparing effect of belimumab [15] is not systematically specified or analysed (due in part to the study design).

Finally, consistent with the scoping review design, it was not the purpose of this analysis to provide aggregate data on the potential steroid-sparing effect of each regimen. We performed a searching with Embase/PubMed filter for RCTs, so we recognize that it is possible that some studies of relevance may have not been identified by using this approach. Similarly, some potentially relevant studies investigating the use of biologics in SLE might have been excluded because the steroid-sparing effect (e.g., steroid tapering protocol and/or cumulative dose) was not quantifiable. Future systematic reviews are required to broaden the spectrum of evaluable works. In conclusion, our study lays the foundation for future research needed to define which patients might benefit most from specific treatment protocols and to establish a clear definition of the steroid-sparing effect guaranteed by new immunosuppressant therapies, aiming to enhance tailored management.

### Supplementary Information

Below is the link to the electronic supplementary material.

Supplementary file1 (DOCX 85 KB)

Supplementary file3 (PDF 57.8 KB)

## Data Availability

Data will be available upon reasonable request.

## Declarations

### Patients and public involvement statement

Patients were not involved in this study.
