# Supplementary figures and images for: Assessing the steroid-sparing effect of biological agents in randomized controlled trials for lupus: a scoping review
Source: Immunol Res. 2024 Mar 9;72(4):538–53. doi: 10.1007/s12026-024-09463-y (PMC11347485; doi:10.1007/s12026-024-09463-y)

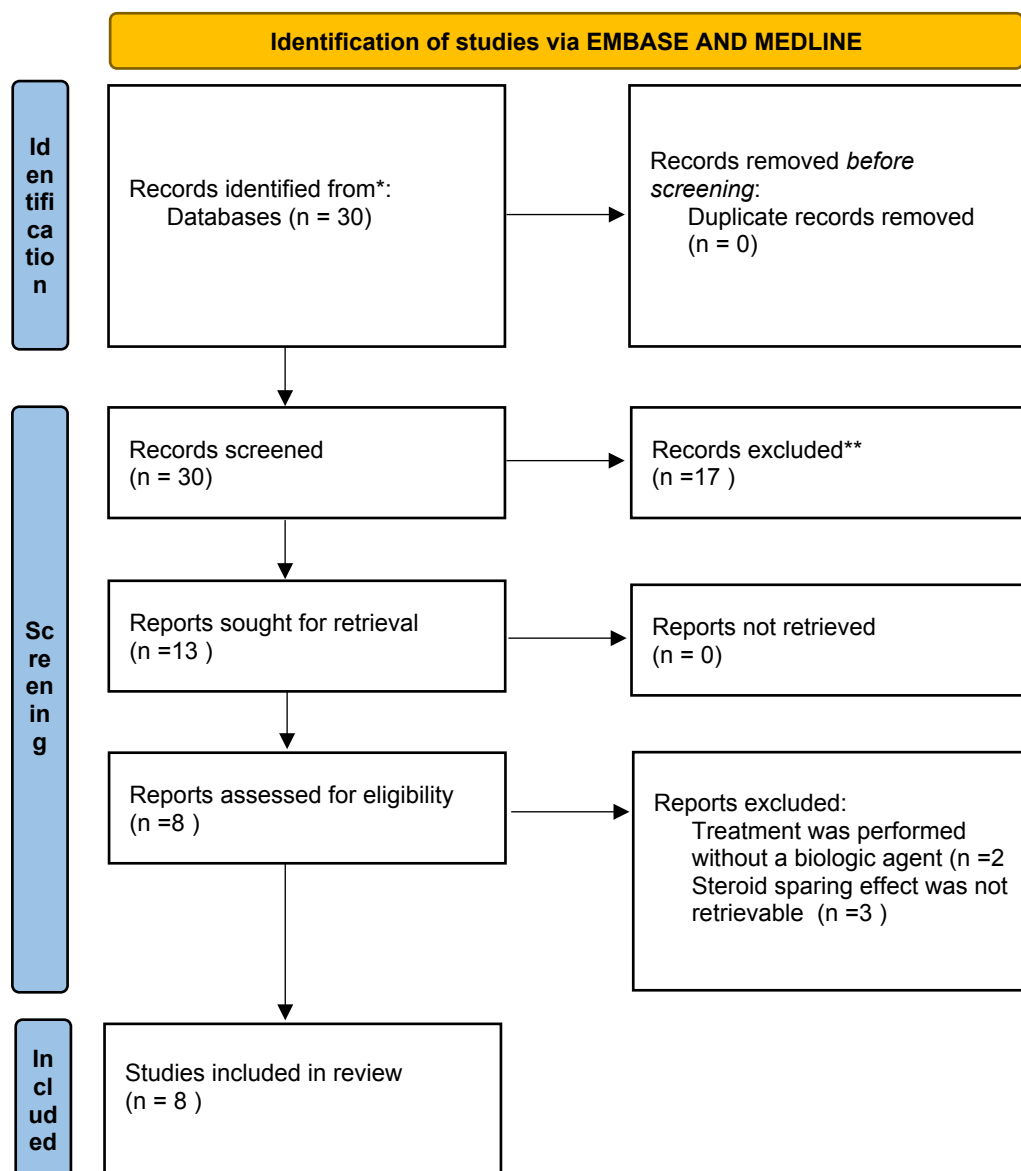

Supplement: Supplementary file 2 — Supplementary file3 (PDF 57.8 KB) [file 12026_2024_9463_MOESM2_ESM.pdf]
